# Supplementary material for: Assessment of label-free quantification and missing value imputation for proteomics in non-human primates
Source: BMC Genomics. 2022 Jul 8;23:496. doi: 10.1186/s12864-022-08723-1 (PMC9264528; doi:10.1186/s12864-022-08723-1)

**Panel A**: Sequence coverage of CA3 detected in our study is marked with bold alphabets in purple color corresponding to >90% of coverage. PTMs of underlined peptide "DIKHDPSLQPWSVSYDGGSAK” are described in more detail in panel B.


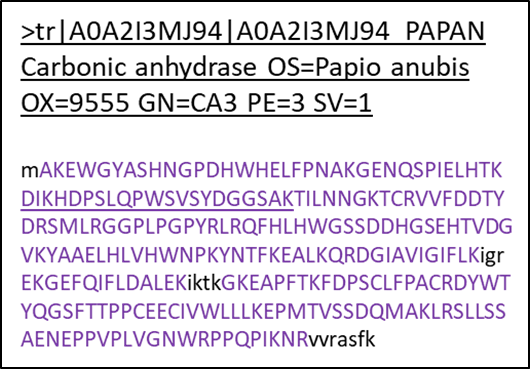


**Panel B**: Various modifications of underlined peptide "DIKHDPSLQPWSVSYDGGSAK” and corresponding MS/MS spectra match with “b” and “y” ions are shown below.

1. Acetylation on K and Magnesium on D


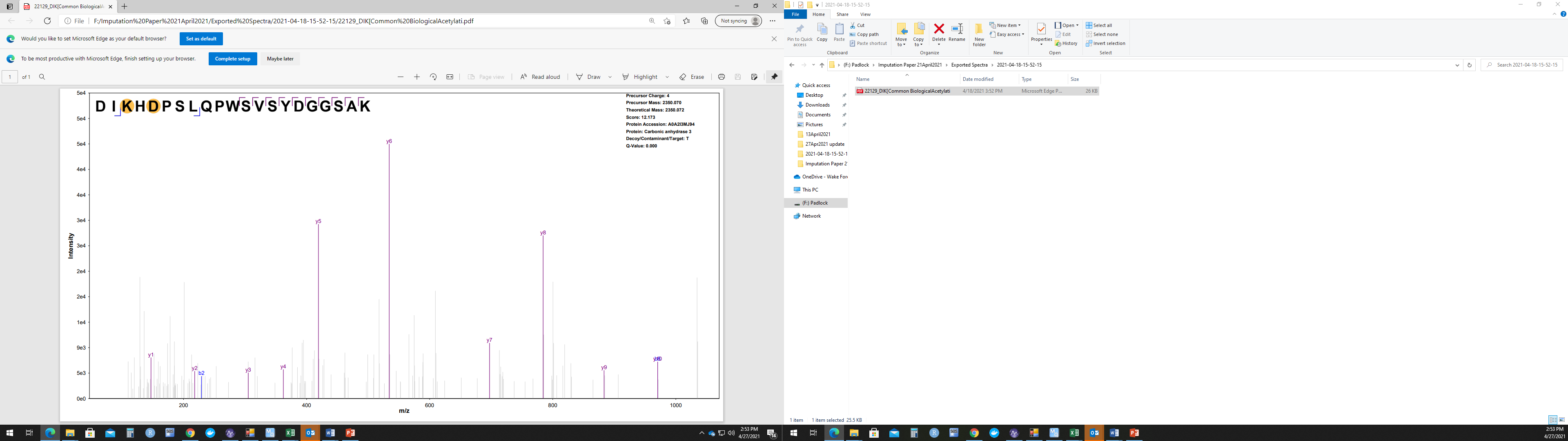


1. **Fe[II] on D**


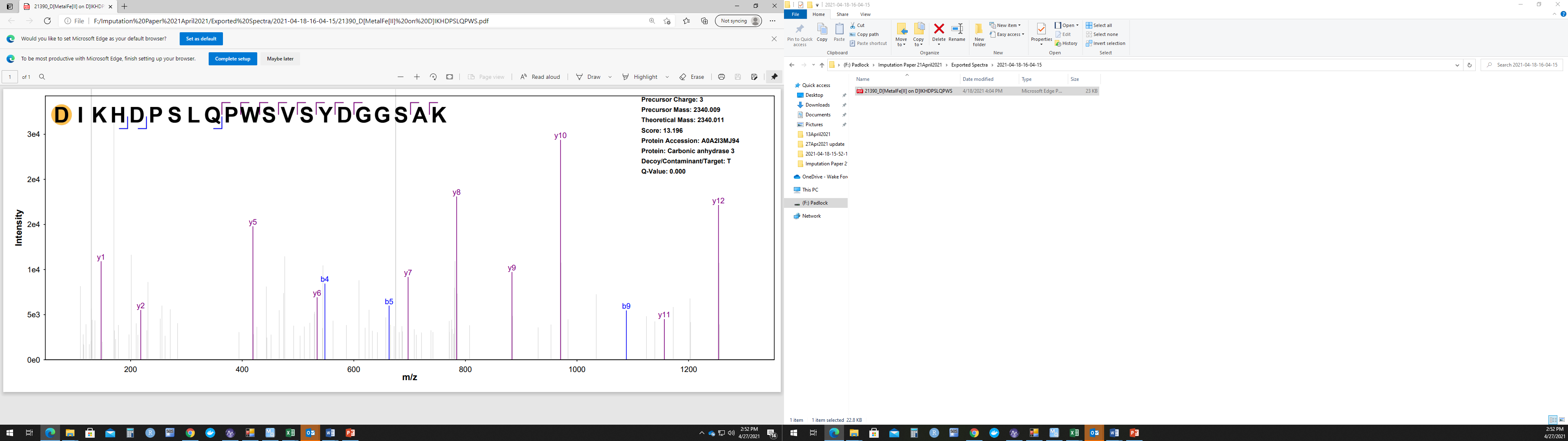

1. **Fe[III] on D**


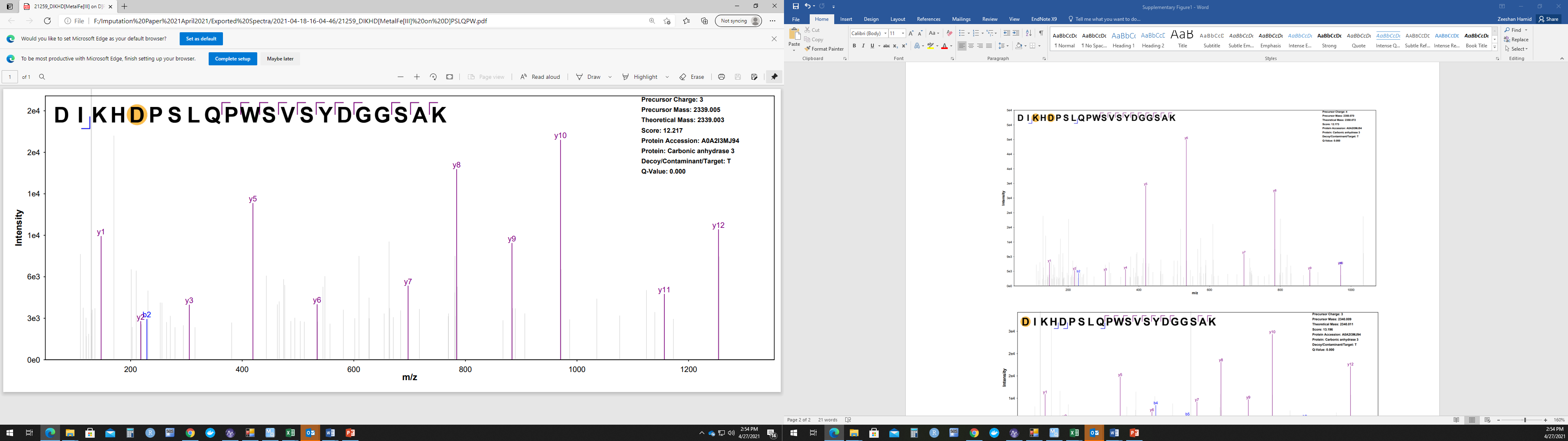

1. **Formylation on K**


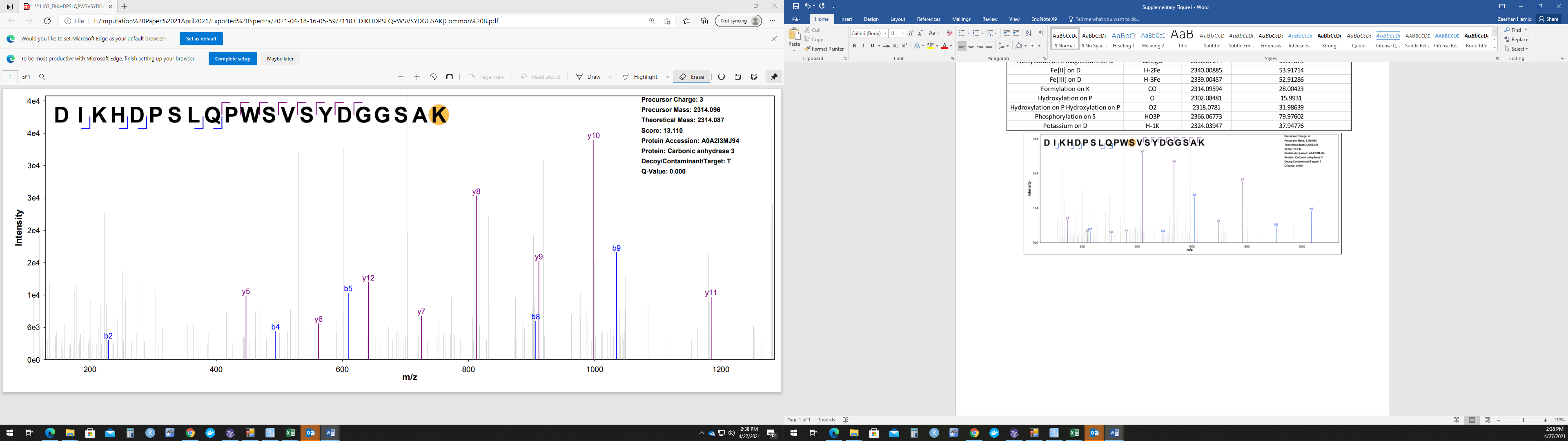


1. **Hydroxylation on P**


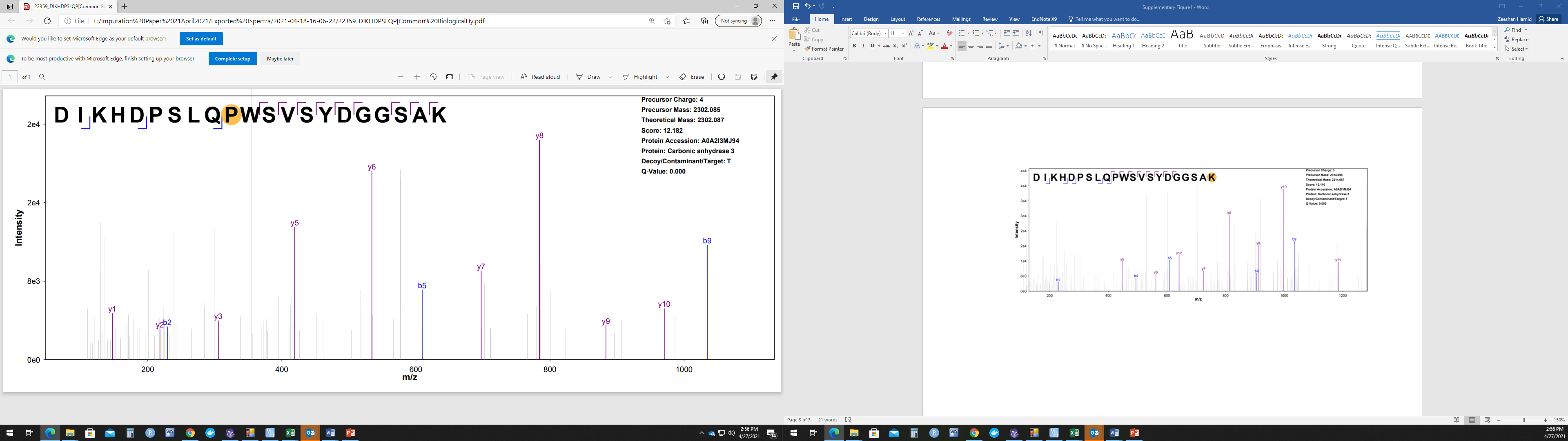

1. **Hydroxylation on P1 P2**


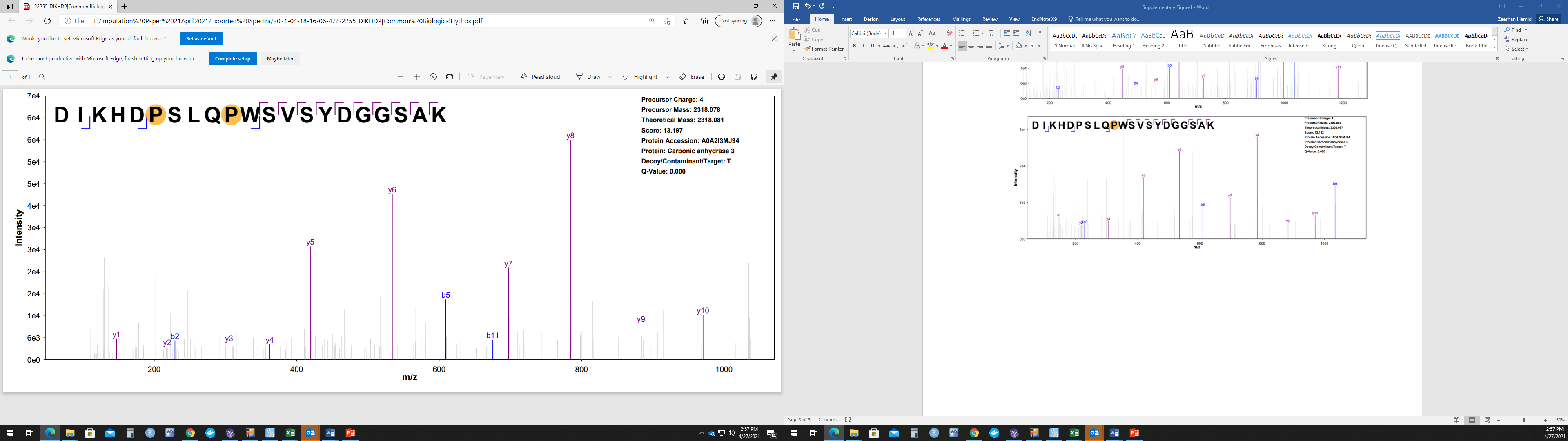


1. **Phosphorylation on S**


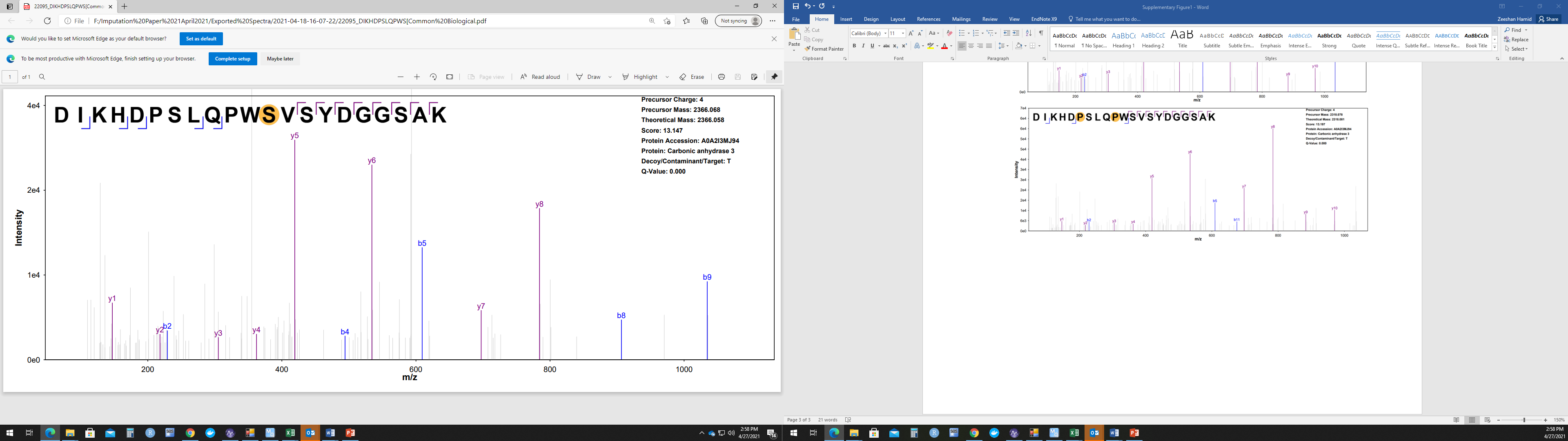

1. **Potassium on D**


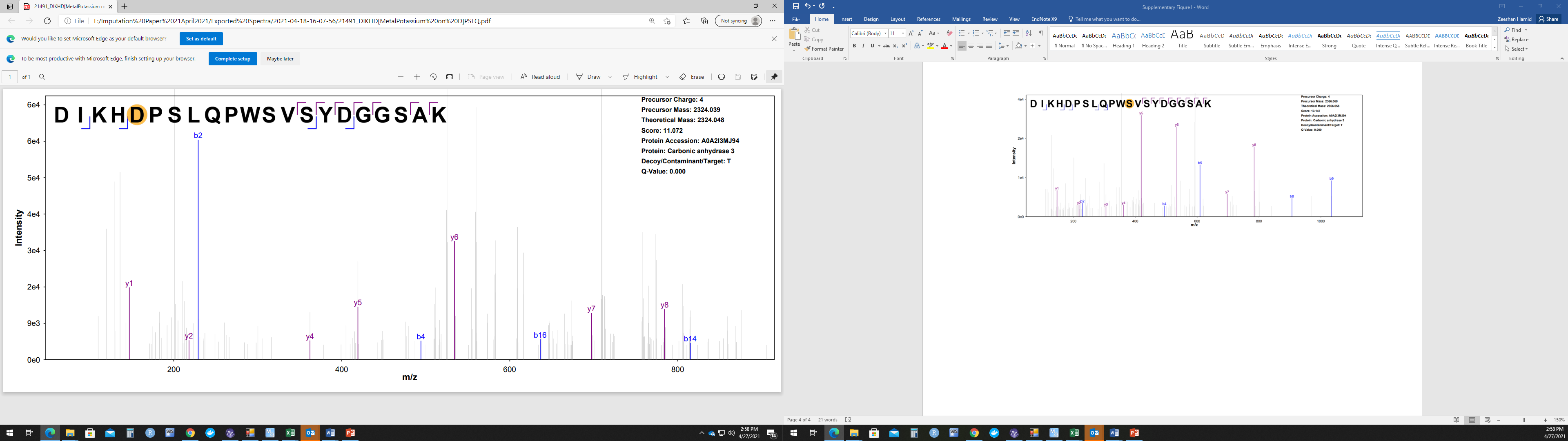

Supplement: Supplementary file 4 — Additional file 4: Supp. File 4. Sequence coverage of reference protein carbonic anhydrase 3 (CA3) observed in our study and MS/MS spectral match of various PTMs identified for reference peptide from CA3. Additional information of co-eluting peptides is also included. [file 12864_2022_8723_MOESM4_ESM.docx]
