# Supplementary figures and images for: Assessment of label-free quantification and missing value imputation for proteomics in non-human primates
Source: BMC Genomics. 2022 Jul 8;23:496. doi: 10.1186/s12864-022-08723-1 (PMC9264528; doi:10.1186/s12864-022-08723-1)

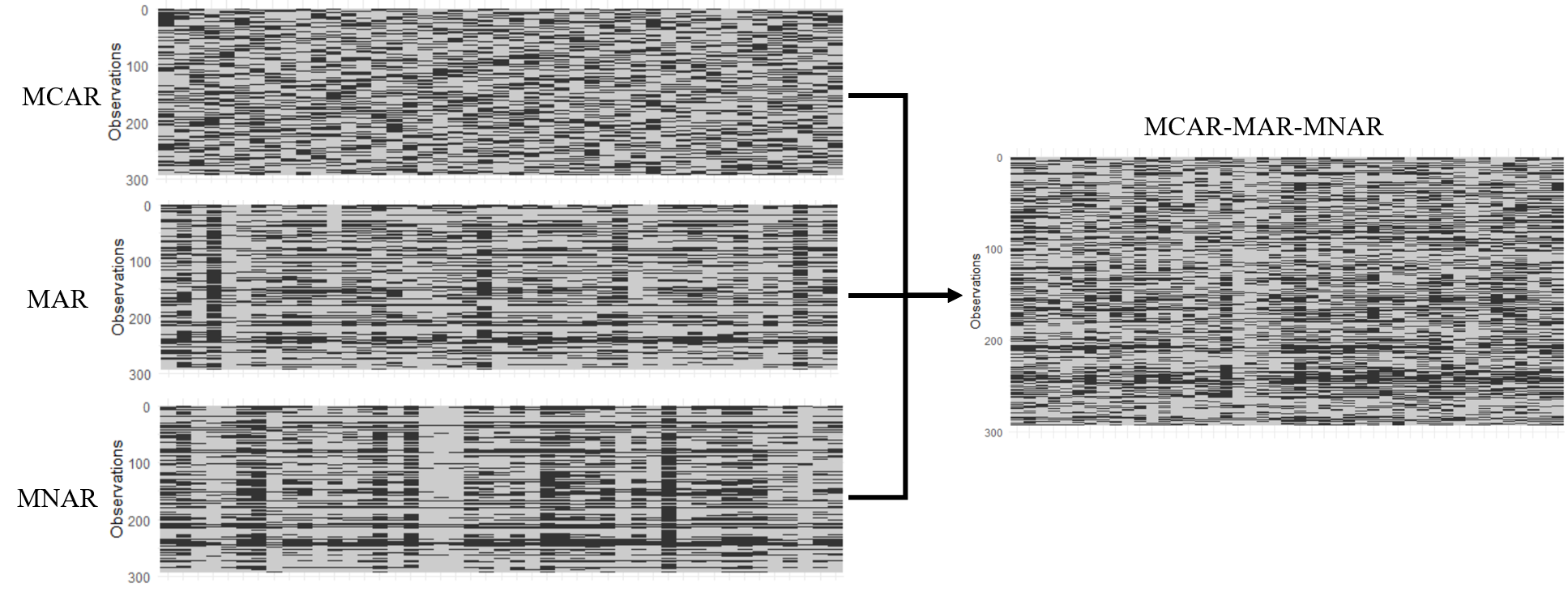

Supplement: Supplementary file 7 — Additional file 7: Supp. Figure 1. Introduction of different types of missingness in the protein list (The original list corresponds to 296 proteins with no missing values in the original dataset). [file 12864_2022_8723_MOESM7_ESM.tif]

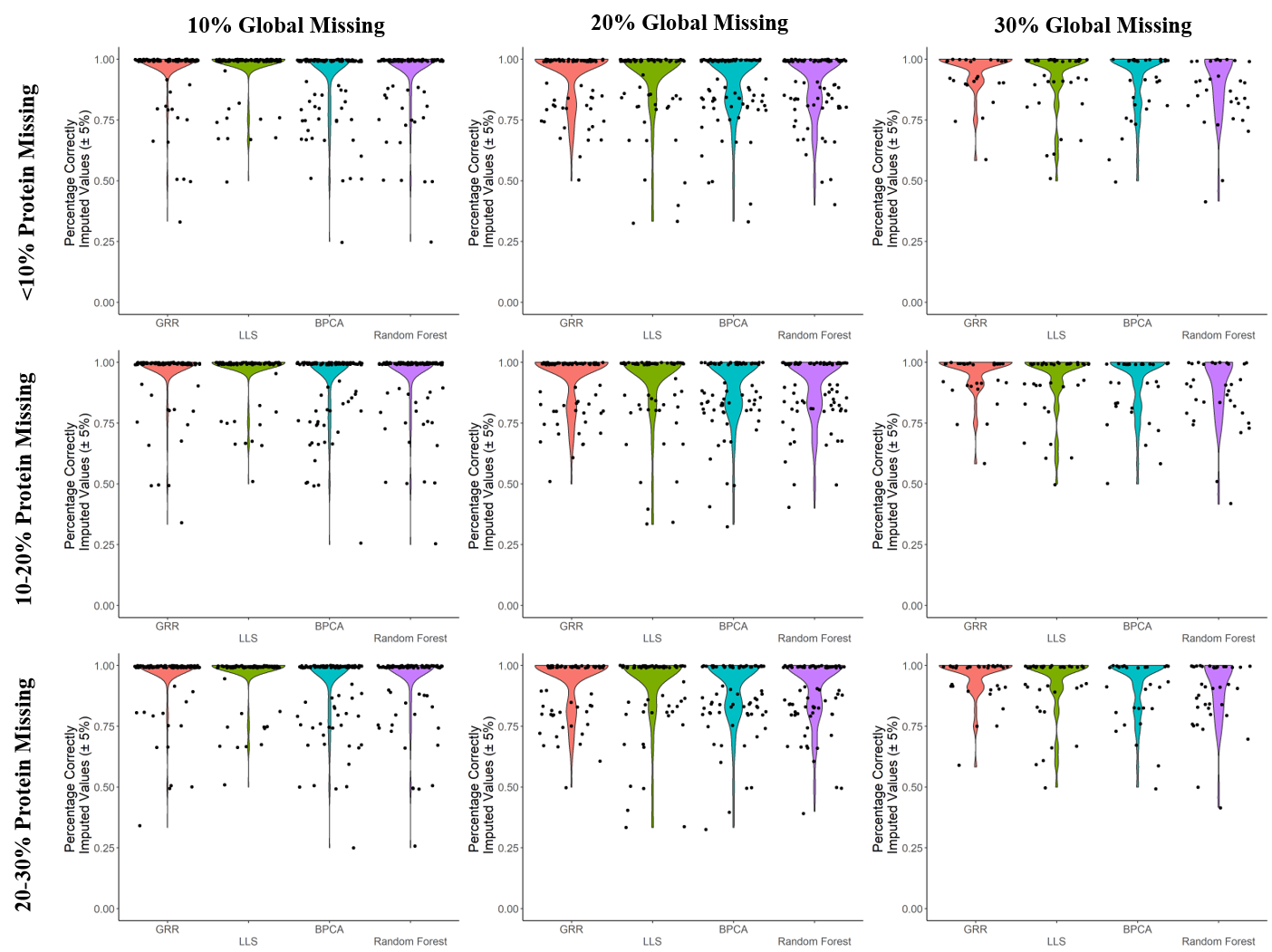

Supplement: Supplementary file 8 — Additional file 8: Supp. Figure 2. Evaluation of four single imputation methods (GRR, LLS, BPCA and Random Forest) at three different levels of missingness (< 10%, 10–20% and 20–30%). [file 12864_2022_8723_MOESM8_ESM.tif]

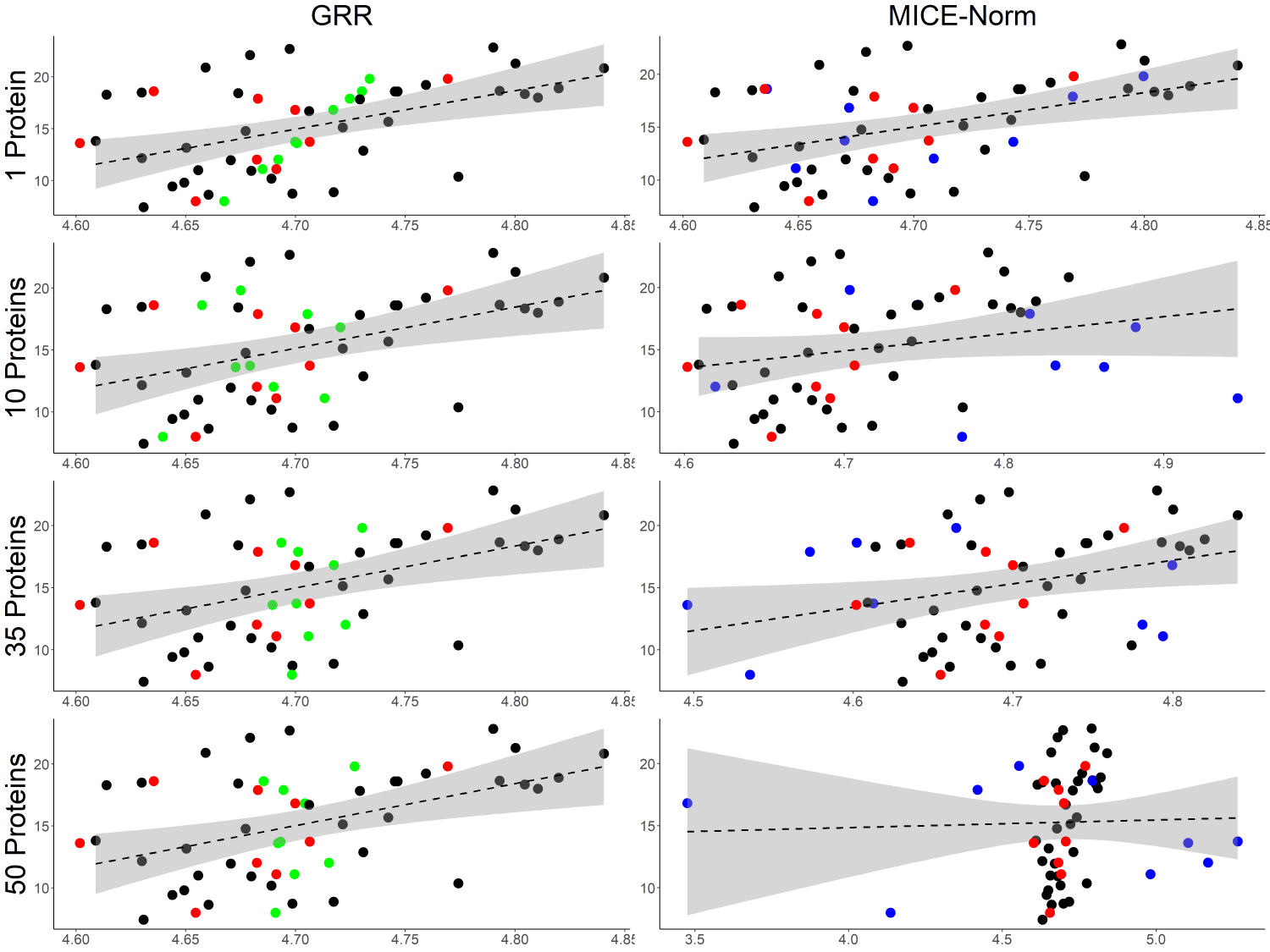

Supplement: Supplementary file 9 — Additional file 9: Supp. Figure 3. Example of the performance of imputation with parametric Single (GRR) and Multiple Imputation (MICE-Norm) methods. X-axis is protein intensity. Y-axis is “P. Anubis” age. Red is truth. Green are values imputed via GRR. Blue are values imputed via MICE-Norm. [file 12864_2022_8723_MOESM9_ESM.tif]

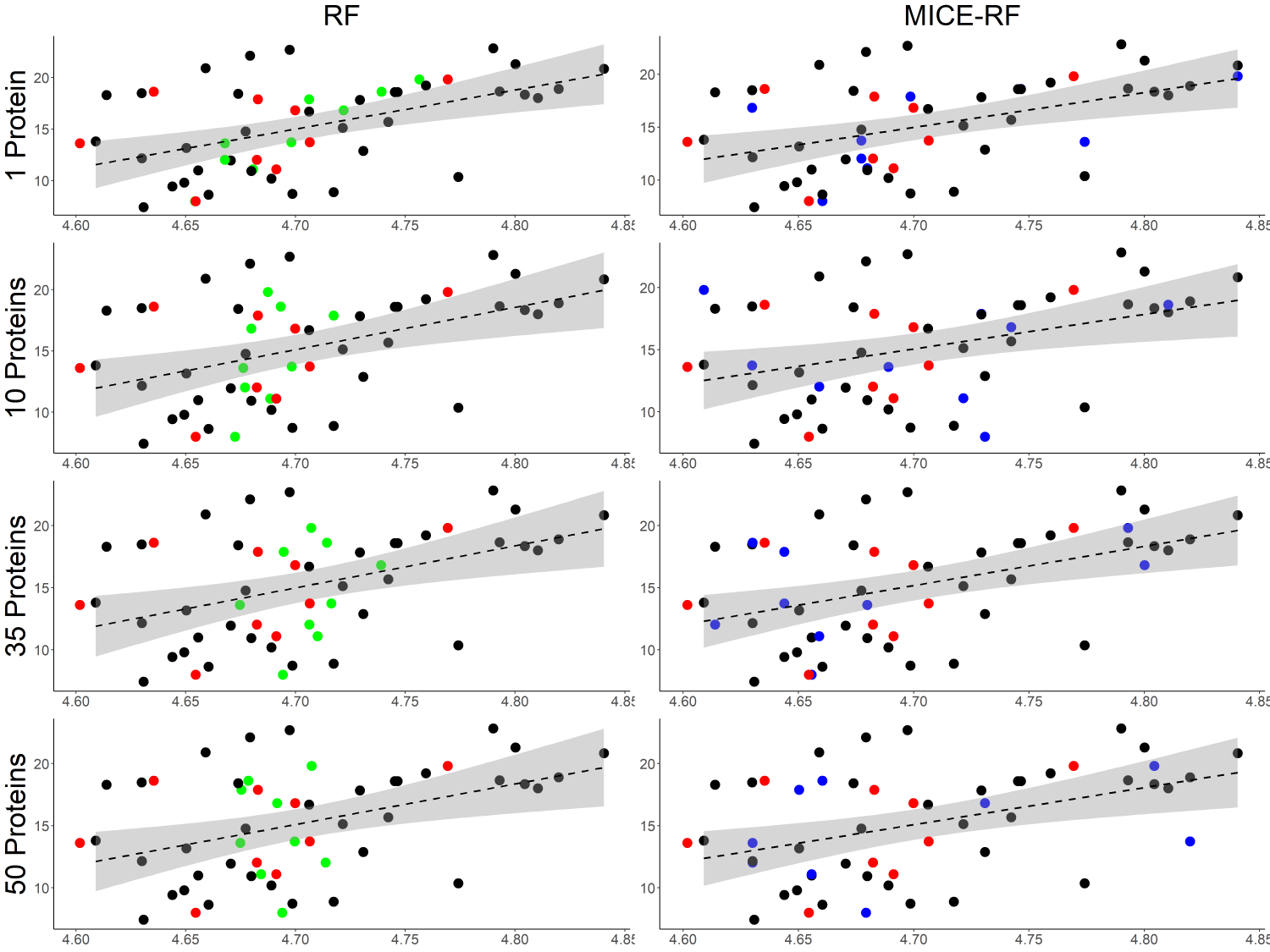

Supplement: Supplementary file 10 — Additional file 10: Supp. Figure 4. Example of the performance of imputation with non-parametric Single (RF) and Multiple Imputation (MICE-RF) methods. X-axis is protein intensity. Y-axis is “P. Anubis” age. Red is truth. Green are values imputed via RF. Blue are values imputed via MICE-RF. [file 12864_2022_8723_MOESM10_ESM.tif]

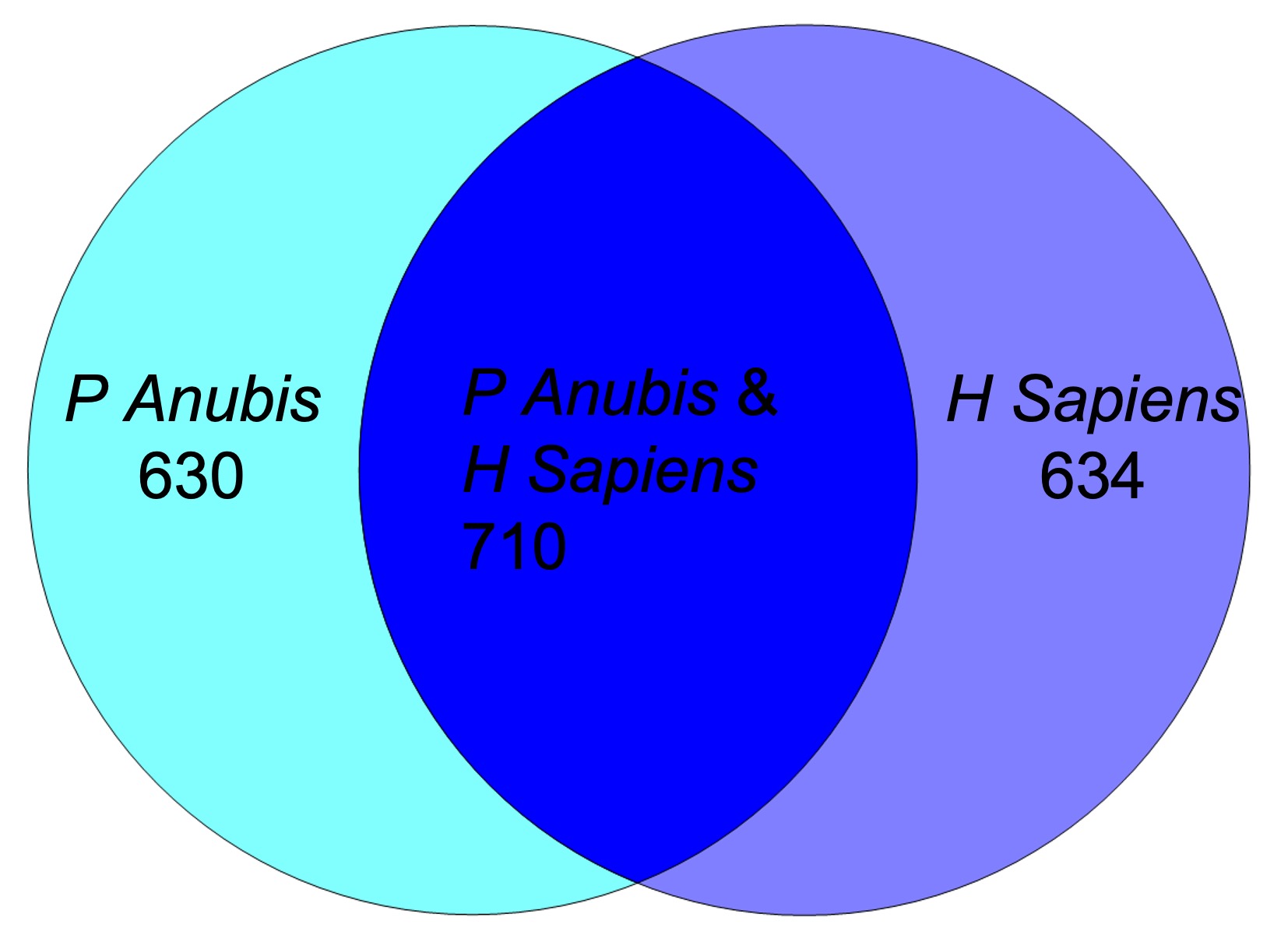

Supplement: Supplementary file 11 — Additional file 11: Supp. Figure 5. Number of proteins observed in a combined proteomics database search specific to each organism (H. sapiens and P. Anubis). [file 12864_2022_8723_MOESM11_ESM.jpg]
